# Reality erosion in digital environments: the concept of digital blur and scale development

**DOI:** 10.3389/fpsyg.2026.1884865

**Published:** 2026-08-14

**Authors:** Onur Oktaysoy, Ethem Topcuoglu, Vurgun Topcuoglu

**Affiliations:** 1 Faculty of Economics and Administrative Sciences, Kafkas University, Kars, Türkiye; 2 Academy of Civil Aviation, Giresun University, Giresun, Türkiye; 3 Faculty of Economics, Administrative and Social Sciences, Istanbul Nişantaşi University, Istanbul, Türkiye

**Keywords:** artificial intelligence, digital blur, digital reality, digitalization, reality perception

## Abstract

**Introduction:**

People are exposed to thousands of videos and images for hours every day. However, the literature provides only limited insight into the impact of AI-generated images and videos on daily life. Driven by advancing technology, these materials influence the human mind and create psychological stress. The source of these psychological tensions lies in situations where the distinction between the real and the artificial is no longer perceived, the sense of reality is lost, and individuals find themselves moving toward a state of uncertainty. This study proposes the concept of “digital blur” in order to conceptualize the reality uncertainty that has emerged with the development of digital technologies and artificial intelligence-supported content production systems, and that has not been adequately explained in the existing literature. The high level of resemblance of digitally produced content to reality makes it difficult for individuals to reliably evaluate not only the accuracy of information but also its reality status, and creates a new area of uncertainty at the epistemic level. This study aims to address this uncertainty not only in the context of misinformation or disinformation, but also through the systematic weakening of the distinction between the real and the artificial, and to fill an important conceptual gap in the literature.

**Methods:**

In the study, scale development procedures were applied to the data collected from two different samples by following the 10 steps suggested by Carpenter. In this context, exploratory factor analysis and confirmatory factor analysis were conducted with SPSS and AMOS programs.

**Results:**

A 14-question scale was developed as a result of factor analyses, discriminant validity, reliability, divergent, convergent, and construct validity. The scale has a two-dimensional structure consisting of Reality Blur and Knowledge and Ethics Blur subscales.

**Discussions:**

In this framework, the study developed an original conceptual framework that explains the transformation of reality perception in the digital age on the basis of Social Information Processing Theory, presented a psychometrically valid and reliable measurement tool to measure this concept, and put forward a new analytical perspective to understand individual and institutional processes regarding the evaluation of digital content.

## 1 Introduction

The rapid progress in digital technologies in recent years has not only increased the speed of production and circulation of information, but has also led to radical transformations in the nature and perception of reality (García-Navarro and Pulido-Martos, 2026). Especially with the development of artificial intelligence, deep learning and generative models, the ability to produce content such as text, images, audio, and video in a way that is extremely close to reality has made the ontological status of information encountered in the digital environment controversial (Chesney and Citron, 2019; Koçbaş, 2025). This situation makes it difficult for individuals to evaluate what is real and what is artificial and creates a new area of uncertainty in the digital information ecosystem (Sandra, 2025). Synthetic content produced in the digital environment not only accelerates the spread of misinformation or deliberate disinformation, but also brings with it multidimensional risks such as forgery, identity manipulation, reputational damage and uncertainty of legal responsibilities (Petratos, 2021). At this point, the main problem faced in the digital age is not only the existence of misinformation, but also the epistemic inability to determine the extent to which information is true (Alatoai and Alshahri, 2025). In other words, the problem is not the accuracy of the content, but the increasing uncertainty regarding the reality status of the content.

In the literature, various conceptual frameworks such as misinformation and disinformation (Lewandowsky et al., 2017; Wardle and Derakhshan, 2017), digital overload (Tarafdar et al., 2015; Abdulkareem et al., 2024), techno-stress (La Torre et al., 2020), algorithmic opacity (Burrell, 2016) and context collapse (Marwick and Boyd, 2011) have been established to explain the problems that arise in digital environments. However, these approaches mostly focus on either the accuracy and production intention of information, the burden on the cognitive capacity of the individual, or the transparency of technological systems (Alsabah et al., 2025). In contrast, the systematic obscuring of the reality reference of digital content and how this is experienced by individuals and institutions is not sufficiently addressed with a holistic conceptual framework in the existing literature (Feliciano-Cestero et al., 2023; Massa et al., 2023). In addition, the relationship of digitally produced content with reality erodes classical understandings of truth and representation and makes individuals' judgments about information more uncertain (Floridi, 2014; Lee et al., 2026). This situation is closely related to the debates on post-truth and epistemic uncertainty. In particular, the high degree of resemblance of digital content to reality can lead individuals to be skeptical not only of false information but also of true information, which can lead to a general erosion of trust (van der Linden et al., 2020).

This study proposes the concept of “digital blur” to fill this conceptual gap. Digital blur refers to the inability of individuals and organizations to consistently and reliably assess the reality status of content encountered in the digital environment as a result of the development of digital technologies and artificial intelligence-supported production tools (Kim et al., 2025). Rather than defining a specific type of content, the concept refers to a structural blur in the conditions of production, circulation, and perception of information in the digital age (Zhao et al., 2026). Unlike the concepts of misinformation or disinformation, digital blur centres on the perceptual and epistemic weakening of the distinction between the real and the artificial, regardless of the accuracy of the content. In this respect, digital blur is not a type of misinformation, but rather an antecedent condition that explains why misinformation has become more effective (Lewandowsky et al., 2017). Moreover, the concept has a multi-layered structure that is not only limited to individual perception processes, but also has an impact on institutional decision-making mechanisms, legal evaluations and ethical judgments (Citron and Pasquale, 2014).

Accordingly, the main purpose of this study is to introduce the concept of “digital blur”, which explains the systematic erosion of reality perception in digital environments on the basis of Social Information Processing (SIP) Theory (Walther, 1992), to the literature, and to develop a valid and reliable scale to measure this concept. In line with this purpose, the study makes three main contributions. First, it proposes a new conceptual framework that explains the transformation of reality perception in the digital age. Second, it develops a psychometrically valid and reliable instrument to measure this concept. Third, it offers a new analytical perspective for understanding individual and organizational processes of evaluating digital content.

## 2 Theoretical and conceptual framework

The Social Information Processing (SIP) Theory (Walther, 1992) forms the basis of this study. While the theory was initially used to explain the way people process impressions and information in computer-mediated communication, today it has gained a place in the literature as an important theory in explaining social media relationships (Khurana and Krishnan, 2025). The theory is frequently used to explain the basic processes of attitudes in computer-mediated communication and the impact of relational development on people (Ramirez, 2007). While Social Information Processing (SIP) Theory was initially used to explain the effects of textual decoding and mutual communication on people, it is now used to explain more phenomena with the development of technology (Marmat, 2022). In this respect, the theory states that people direct their behaviors, perceptions, and relationships by questioning, coding, and making sense of the texts and visuals they perceive (Tidwell and Walther, 2002). The theory argues that since humans are social beings, building relationships is not only possible in face-to-face communication environments but also in different environments (Marmat, 2022). Floridi (2014) emphasizes that in digital environments, information is not only represented but also reproduced. This situation increasingly obscures the reality reference of digital content and makes individuals' evaluation processes of information more complex. The concept of digital blur explains the reflection of this structural transformation at the level of individual perception. In addition, the increasing role of algorithmic systems leads to a decrease in transparency in the production and distribution of information. In order to explain the hesitations experienced in these and similar cases, this study defines the concept of digital blur by utilizing SIP Theory.

### 2.1 Definition and scope of digital blur concept

The quality of content produced and circulated in the digital environment, especially with the development of artificial intelligence and generative models, has revealed a new problem area that goes beyond classical information evaluation criteria (Vartiainen et al., 2025). Studies on synthetic media and deepfake technologies reveal that digital content can be produced in a way that is extremely close to reality, making it difficult for individuals to evaluate content (Vaccari and Chadwick, 2020). These developments make not only the accuracy but also the reality status of the information encountered in the digital environment controversial and, as mentioned earlier, weaken individuals' capacity to evaluate what is real and what is artificial (Bilişli et al., 2024).

The emergence of the concept of digital blur is driven by multiple structural transformations. First, the ability of artificial intelligence-supported production technologies to produce content that is extremely close to reality leads to a perceptual weakening of the distinction between real and artificial (Chesney and Citron, 2019). Secondly, the increase in the speed of information production and the diversity of sources in the digital environment complicate the evaluation processes regarding the source and reliability of information (Lewandowsky et al., 2017). Third, the increasing role of algorithmic systems in content production and distribution processes reduces the transparency of how knowledge is produced and by whom, which weakens individuals' epistemic trust (Burrell, 2016). Finally, the intertwining of different types of content and forms of representation in the digital environment makes the reality reference of information increasingly ambiguous (Floridi, 2014). This multi-layered transformation paves the way for the erosion of established criteria for evaluating information in the digital environment and the emergence of a new state of uncertainty.

This study proposes the concept of “digital blur” to the literature to explain this state of uncertainty. Digital blur refers to the situation in which the reality status of information produced in the digital environment cannot be evaluated by individuals and institutions in a consistent, reliable and stable manner due to the fact that digital technologies and artificial intelligence-supported content production systems can produce outputs that are very close to reality as a result of the development they have achieved, and thus the distance between real and fake is narrowed and broken. Rather than defining a specific type of content, this concept points to a structural blur in the conditions of production and perception of information in the digital age. This uncertainty undermines individuals‘ assessments of the accuracy, source, and intent of digital content, and renders institutions' information-based decision-making, attribution of responsibility, and accountability processes fragile. Digital blur, which is characterized by systematic difficulty in assessing the accuracy, source, and authenticity of digital content, can be operationalized as perceptual and cognitive uncertainty in the processes of distinguishing, verifying, and establishing trust in the authenticity of digital content. This definition allows digital blur to be treated as a measurable construct and enables the concept to be subject to empirical research.

The scope of the concept is limited to the evaluation processes regarding the reality reference of information produced in the digital environment. In this framework, digital blur is not directly related to the accuracy or algorithmic performance of technical systems, but rather focuses on how individuals perceive the content produced by these systems (Hynek et al., 2025). In this respect, the concept is positioned as a perceptual and epistemic condition rather than a technical problem. The scope of the concept is not limited to individual perception processes. Digital blur has a multi-layered structure that also affects institutional decision-making, legal evaluation, and ethical judgment processes. The inability to distinguish artificially generated content from real content makes it difficult to determine the nature of evidence, obscures the attribution of responsibility, and blurs the boundaries of accountability mechanisms (Citron and Pasquale, 2014). In this respect, digital blurring provides a conceptual framework that not only affects the effects of digital technologies on the information ecosystem but also the functioning of the legal and administrative order.

The concept of digital blur proposed in this study is primarily addressed through the perceptual and cognitive evaluation processes of individuals toward digital content. However, it is also recognized that the concept may have broader implications on information-based decision-making processes and trust relationships in digital environments. This limitation aims to clarify the analytical framework of the concept and increase its measurability.

### 2.2 Differentiation of digital blur from similar concepts

In the digital transformation literature, various conceptual frameworks have been developed to explain the destructive effects of digital technologies on individuals, organizations, and society (Feliciano-Cestero et al., 2023). Concepts such as misinformation, disinformation, digital overload, technostress, algorithmic opacity, and context collapse offer important contributions to understanding the different problem areas that arise in the digital environment (Tarafdar et al., 2015; Mortazavi Ravari et al., 2016; La Torre et al., 2019; Vaccari and Chadwick, 2020; Petratos, 2021; Bilişli et al., 2024; Alsabah et al., 2025; Olesen, 2025). However, each of these concepts focuses on a specific phenomenon or outcome and is limited in explaining the systematic weakening of the distinction between the real and the artificial at the perceptual and epistemic levels. This section analytically distinguishes the concept of digital blur from these approaches.

The misinformation and disinformation literature focuses on the accuracy and production intention of content circulating in the digital environment (Wardle and Derakhshan, 2017). These approaches address misinformation or deliberate distortion of information as the main problem and propose solutions through content verification, media literacy, or platform regulation (Adams et al., 2023). Digital blur, on the other hand, centers on the inability of individuals and institutions to reliably distinguish the reality status of digital content, regardless of the accuracy of the content or the intention of its production. In this respect, digital blur is positioned as an antecedent condition that explains why misinformation can be so effective, rather than misinformation itself (Lewandowsky et al., 2017).

Digital overload and techno-stress approaches focus on the cognitive, emotional, and physiological burdens that individuals are exposed to digital technologies (Tarafdar et al., 2015; La Torre et al., 2019). This literature explains that individuals' decision-making quality decreases and stress levels increase as a result of exposure to too much information. However, digital blur focuses on the ontological quality of information rather than its quantity. In other words, the problem is not “too much information” but the inability to distinguish whether the information encountered is real or artificial. Therefore, digital blur is conceptually distinct from overload or stress-based explanations.

The concept of algorithmic opacity emphasizes the lack of transparency in the decision-making processes of artificial intelligence and machine learning systems and the inability of users to understand how these systems work (Burrell, 2016; Guo et al., 2025). This approach provides an important framework for discussions on algorithmic accountability and fairness. However, while algorithmic opacity focuses on the inner workings of technological systems, digital opacity focuses on the effects of the outputs of these systems on the perception of reality (Olesen, 2025). Hence, digital opacity explains the blur in the conditions under which outputs are perceived and evaluated, rather than the problems of technical transparency.

The concept of context collapse addresses the intertwining of different social contexts, especially in social media environments, and its effects on communication processes (Marwick and Boyd, 2011). This approach makes important contributions to understanding the social dimensions of digital communication. However, context collapse focuses mainly on the blur over social roles and communication norms and does not directly address the weakening of the epistemic distinction between reality and artifice (Loh and Walsh, 2021). Digital blur, on the other hand, describes a more fundamental level of uncertainty about the reality status of digital content, independent of social contexts.

Finally, studies on synthetic media and deepfake technologies mostly examine the effects of artificially generated content on individuals and society from ethical, political, and security perspectives (Chesney and Citron, 2019; Vaccari and Chadwick, 2020; Yildirim and Yolcu, 2022). While this literature makes the consequences of digital blur visible, it does not unify these consequences under an overarching conceptual framework. The concept of digital blur aims to explain how these technologies systematically erode the perception of reality, rather than the singular effects of synthetic content.

In this framework, digital blur is not a renaming of concepts in the existing literature, but a conceptualization of a new perceptual and epistemic situation that emerged with the development of digital technologies. The concept offers a high-level analytical framework to explain the transformation of the reliability of information, perception of reality, and decision-making processes in the digital age, and in this respect, it systematically differs from existing approaches. Table 1 below summarizes this distinction.

**Table 1:** Systematic differentiation of the concept of digital blur from related concepts.

Concept	Focus point	Level of analysis	Fundamental difference from digital blur
Misinformation	Accuracy of the content	Content-based	Digital blur focuses on the inability to distinguish the reality status of content, regardless of whether it is true or false.
Disinformation	Deliberate misleading	Content and intention-based	Digital blur is not centered on intention, but on perceptual and epistemic uncertainty.
Digital overload	Quantity of information	Cognitive capacity	Digital blur problematizes the ontological quality of information, not its quantity.
Technostress	Technology-induced stress	Psychological	Digital blur is not a stress response, but a distortion in the perception of reality.
Algorithmic opacity	Transparency of systems	Technical/system level	Digital blur focuses on how the output is perceived rather than how the algorithm works.
Context collapse	Merging social contexts	Communicative	Digital blur explains the blurring of the real-artificial distinction regardless of social context.
Synthetic media/deep fake	Artificial content generation	Technological	Digital blur conceptualizes not the technologies themselves, but the perceptual outcome they create.
Digital blur (suggested concept)	Blurring the real-artificial distinction	Ontological and epistemic	Explains the inability to reliably assess the reality status of information in the digital age.

### 2.3 Digital blur sub-dimensions

Digital blur is too complex to be reduced to a one-dimensional or purely individual perception. The development of digital technologies and artificial intelligence-supported content production systems causes the distinction between real and artificial to weaken simultaneously on different planes (Liu and Li, 2025). Therefore, digital blur should be considered as a multidimensional conceptual structure extending from individual perception processes to institutional decision mechanisms. In the existing literature, discussions on the effects of digital content indirectly touch upon some of these dimensions, but fail to unify them under a holistic framework (Floridi, 2014; Lewandowsky et al., 2017).

This study argues that digital blur can be conceptualized in terms of two interrelated but analytically separable dimensions.

Information and Ethical Blur: Information blur refers to the weakening of evaluation processes regarding the accuracy, source, and validity of information encountered in the digital environment. The high degree of realism in digital content leads individuals to question not only the accuracy of specific pieces of information but also the reliability of digital information in general (Pennycook and Rand, 2019). At the same time, information blurring refers to the blurring of the line between what is ethically acceptable and unacceptable due to the technical capabilities offered by digital technologies. The high degree of realism in artificially generated content complicates the distinction between “what is possible” and “what should be done,” rendering ethical decision-making processes fragile (Floridi et al., 2018). This dimension is of critical importance for digital leadership, corporate governance, and public regulations. Information and ethical blur represents the normative dimension of digital blur and focuses on what is right, what the boundaries should be, and alignment with values. This dimension encompasses the uncertainty of information's epistemic value, independent of the spread of misinformation. When considered in the context of social information processing theory, it is seen that people do not always need to come face to face to convey information and thoughts, even a sign is enough to tell the situation. The smiley face emoji in a message sent by a person helps us understand many things about the person's situation without saying anything. Likewise, a visual or video made through artificial intelligence tells people many things and allows them to make inferences. However, in the current situation, the information is altered in an unethical way and expresses a situation that the person receiving the message will misunderstand. Therefore, when processing social information, the person moves away from contextual social norms and lacks past life clues about information processing and experiences blurring. This blurring may cause the individual to adopt unethical behaviors and perceive these behaviors as a social norm of life (Tidwell and Walther, 2002).

#### 2.3.1 Reality blur

This dimension refers to the weakening of the perception of the reality reference of the content produced in the digital environment. The fact that texts, images, and videos created by artificial intelligence-supported generative systems become indistinguishable from real content makes it difficult for individuals to evaluate the ontological status of the content they encounter (Vaccari and Chadwick, 2020). This dimension is associated with the proliferation of ways of representing reality and the blurring of boundaries between these representations, rather than the disappearance of reality. Reality blur constitutes the most fundamental and triggering dimension of digital blur. According to SIP Theory, due to the lack or inaccuracy of available cues in the delivery of a message or perceived value, the receiver may attribute unrealistic negative characteristics to the sender. While SIP Theory envisages that the messages transmitted within the scope of SIP Theory should be limited as true or false, in reality blur, the message does not have the desired effect because truth and falsehood are intertwined and it may take a very long time to perceive the real message. In this case, it leads individuals into a confusion. In short, while SIP theory defines the interaction model that should be, reality blur represents the most undesirable situation within the model (Marmat, 2022).

#### 2.3.2 Relationship between sub-dimensions

These two sub-dimensions, which are defined and framed above, are not independent of each other, but rather mutually nourishing and reinforcing. Reality blur triggers information and ethical blur, which in turn leads to the blur of information and ethical boundaries. Therefore, digital blur should not be considered as a singular perception problem, but as a multi-layered transformation process regarding the production, evaluation, and management of information in the digital age.

### 2.4 Individual, organizational and social consequences of digital blur

Digital blur, as mentioned earlier, is not only a perception problem regarding the quality of digital content, but can produce multi-layered results that affect the information-based judgments of individuals, the decision-making processes of organizations, and the trust-based functioning of societies. The inability to distinguish between the real and the artificial weakens the established mechanisms for evaluating information in the digital environment and leads to mutually reinforcing results at different levels of analysis (Ramaul et al., 2025). At this point, it would be appropriate to mention the possible consequences of digital blur at individual, organizational, and societal levels.

### 2.5 Digital blur at the individual level

The most prominent consequence of digital blur at the individual level may be the weakening of individuals‘ epistemic trust in digital content. Uncertainty about whether the information encountered in digital media is real or artificial can make individuals' information evaluation processes more cautious, but this caution may result in indecision, cognitive fatigue, and trust erosion over time (Lewandowsky et al., 2017). In particular, the high degree of resemblance of AI-generated content to reality can invalidate individuals‘ intuitive judgments of accuracy and reduce the effectiveness of cognitive shortcuts (Pennycook and Rand, 2019). This process may lead individuals to not only become more open to misinformation but also to develop a skeptical attitude toward accurate information. This situation, referred to as “generalized suspicion” in the literature, holistically erodes individuals' trust in the digital information ecosystem and weakens their capacity to make rational decisions based on information (van der Linden et al., 2020). In this context, digital blur creates a ground that can be associated with consequences such as information fatigue and a decrease in decision quality at the individual level.

### 2.6 Digital blur at the organizational level

At the organizational level, digital blur can directly affect the reliability of information-based decision-making processes. Uncertainty about the reality status of data obtained from digital sources increases the level of epistemic risk in managers' strategic decisions and makes decision processes more fragile. Especially in organizations where artificial intelligence-supported analysis systems have become widespread, the lack of clarity about the extent to which decisions are based on human judgment and algorithmic outputs weakens responsibility and accountability mechanisms (Kellogg et al., 2020). However, digital blur also has the potential to have significant consequences for organizational trust and corporate reputation. Indeed, inaccurate or misleading digital content influencing organizational decisions or the circulation of fake content produced on behalf of the organization may damage the credibility of organizations in the eyes of stakeholders (Chesney and Citron, 2019). This shows that digital blur is not only a technical risk for organizations, but also an area of strategic and managerial vulnerability.

### 2.7 Digital blur at the social level

The most important consequence of digital blurring at the social level is the destruction and erosion of information-based trust relations at every point of social spheres of influence. The systematic questioning of the authenticity of content produced in the digital environment makes it difficult to conduct public debates on a common ground of reality. This weakens social consensus processes and paves the way for the fragmentation of the public sphere (Habermas, 2006; Vaccari and Chadwick, 2020). In addition, digital blur can also create new problem areas in terms of legal and normative regulations. Issues such as the evaluation of the evidentiary quality of artificially produced content, the perpetrator of the crime, and the inability to determine responsibility may cause existing legal frameworks to be insufficient (Citron, 2019). In this context, digital blur should be considered not only as a byproduct of technological transformation but also as a social phenomenon that requires rethinking in the fields of law, ethics, and governance.

The above-mentioned individual, organizational, and social consequences of digital blur are not independent from each other in terms of the conceptual framework. The weakening of individuals' trust in digital information is reflected in organizational decision-making processes, and the uncertainties experienced at the organizational level make social trust relations even more fragile. This interplay reveals that digital blur should be addressed not as a singular problem area in terms of its effects, but as a multi-level and systemic transformation process.

### 2.8 The necessity of scale development for the concept of digital blur

The concept of digital blur offers a new conceptual framework to explain the multi-layered uncertainty that emerges in the evaluation processes regarding the reality status of information in the digital age. However, for a concept to gain a place in the literature and be subject to empirical research, it is possible not only to define it theoretically but also to transform it into a measurable structure. In this context, the systematic examination of digital blur requires the concept to be supported by a valid and reliable measurement tool. When the existing literature is examined, it is seen that various scales have been developed to measure the problems that arise in digital environments. These scales mostly focus on specific phenomena such as exposure to misinformation, digital overload, techno-stress, or attitudes toward technology use (La Torre et al., 2019; Abdulkareem et al., 2024). However, it is noteworthy that there is no holistic measurement tool to directly measure perceptual and epistemic uncertainty regarding the reality status of digital content. This constitutes an important gap that may lead to the concept of digital blur remaining only at the theoretical level and not being empirically tested.

The development of a scale for measuring digital blur is important in more than one way. Firstly, such a measurement tool will enable the quantitative determination of the level of uncertainty experienced by individuals in their evaluation processes toward digital content. Secondly, it will make it possible to empirically examine the consequences of digital blur at individual, organizational, and societal levels. Thirdly, it will pave the way for analysing the relationships between digital blur and trust in information, decision-making processes, and information evaluation mechanisms in the digital environment. In addition, the multidimensional nature of digital blur shows that this concept cannot be evaluated with simple single-item measurements. Uncertainties related to reality perception, information reliability, and evaluation processes emerge as interrelated but representing different aspects. Therefore, measuring digital blur necessitates the development of a multi-item and psychometrically tested scale that can reflect the different dimensions of the concept.

In this framework, in order to empirically examine the concept of digital blur and to make it a measurable structure in the literature, this study aims to develop a scale to measure digital blur. In the scale development process, an item pool was created based on the theoretical framework of the concept, and this structure was tested through validity and reliability analyses. In the next section, the methodological process followed in the development of the digital blur scale is presented in detail.

## 3 Method

### 3.1 Sampling and generalization

In order to conduct the research in accordance with scientific validity and ethical principles, ethical approval was obtained from the Kafkas University Social Sciences Ethics Committee via Decision No. 95-23, dated February 13, 2026. Interviews with 11 experts regarding content validity were conducted between February 14, 2026, and February 15, 2026. Preliminary test data were collected via convenience sampling from a group consisting of teachers and academics working in Giresun and Kars between February 16, 2026, and February 28, 2026. The primary reason for the pre-test sample consisting of teachers and academics was to ensure the scale was measured using a sample with a certain level of education and to develop a more reliable scale. To collect the data, online surveys were distributed and collected via WhatsApp groups. While the plan was to reach at least 100 participants for the pre-test, the survey was concluded once the number reached 162. For the main sample, data was collected between March 2, 2026, and March 14, 2026, in Giresun, Istanbul, and Kars using convenience sampling among students, public sector employees, and a more general group. A total of 421 participants were reached for the main test, with students constituting the majority of the group (53%). Students were selected for the main test because they lack the necessary equipment and life experience to adequately assess the videos and visuals they are exposed to on social media. The fact that the pre-test collected data from individuals with a higher level of knowledge than the general public, while the main test consisted primarily of students, limits the representativeness of the sample and may restrict the generalizability of the findings to a broader population. The generalizability analysis was performed with the cross-validation prediction ability test (CVPAT) analysis over Smart-PLS. The CVPAT test provides information about the generalizability of the sample through a naive model (indicator mean) that provides minimum prediction accuracy and a robust model (linear model) that produces robust predictions. As a result of the analysis, it was determined that the variables in each model provided generalizability with a *p*-value less than 0.05 (Sharma et al., 2023).

### 3.2 Scale development method

For the development of the scale, a ten-step procedure was created by adapting the steps suggested by Carpenter (2018). Most of the steps of the procedure will be applied for both pretest and main test.

Step 1. Identification of appropriate scale items and sub-dimensions by researching the literature.

a. Obtaining expert opinion for item expressions and conducting content validity,

Opinions were received from 11 experts who have various previous works on digitalization, digital culture, digital transformation and digital leadership. Of the respondents, four were women, and seven were men, and men had a more predominant participation in the research. Four of the experts are professors, three are associate professors and four are assistant professors at various universities in Turkiye. Content Validity Ratio (CVR) method proposed by Ayre and Scally (2014) will be used for the content validity of expert opinions. In this method, the calculation will be made in accordance with the formula below.

CVR: Content Validity Ratio

Ne: The number of experts who believe it is necessary

N: The number of experts

Experts assign scores to each item as “−1 = ” not necessary “, 0 = ” useful, but not essential “, +1 = necessary”. For the sample of 11 experts in total, the ratio of those who agree that the items are necessary should be 0.818, and the CVR critical exact values ratio should be 0.636. Items that do not meet these conditions will be removed from the question pool.

b. Pre-testing with at least 100 people and finalizing the scale items,

In accordance with the steps recommended by Carpenter (2018), at least 100 people should be reached within the scope of the pre-test. Ethics committee permission was obtained before starting the study, and the survey was started after the Ethics Committee Decision was obtained. All processes were carried out in accordance with the Helsinki Declaration criteria. In order for the subject to be understood and the quality of the data to be obtained to be of the desired quality, it was ensured that the people who would participate in the pre-test were composed of teachers and academicians with at least a bachelor's degree. Teachers and academics were specifically selected because they both shape the next generation and live in an integrated way with the current world of life. The questionnaire forms were shared digitally with the teacher groups through a convenience sampling method. Before answering the questionnaires, participants were asked to fill in the consent form digitally, and after the consent form was filled in, the questions were made available. The data collected from the participants within the scope of the pre-test were subjected to normality, correlation, exploratory and confirmatory factor, discriminant validity and parallel analysis to improve the scale.

Step 2. Establish sampling criteria for the actual test.

For the actual test, the questionnaire will be digitally administered to a group of public employees, students, and the general public. The main criterion for selecting participants was the availability of social media accounts. Before answering the questionnaires, the participants were asked to fill in a digital consent form, and after filling in the form, the questions were made available. To determine the minimum number of participants, the formula was determined by Cochran (1977) was used with a 95% confidence interval and a 5% error rate. In the calculation made in this context, it was determined that at least 383 people should be reached.

Step 3. In order to examine the quality of the data, normal distribution, erroneous data control, and correlation analysis will be performed with the IBM SPSS program.

Step 4. Exploratory factor analysis will be applied to the data with the IBM SPSS program to determine the factor structure.

Step 5. Applying Parallel Analysis with syntax codes (O'Connor, 2000) via the IBM SPSS program to confirm the identified factor structure, checking the variances explained.

Step 6. Removing the poorly loaded factor items in the factor structure, ensuring that each factor structure consists of at least three items, examining the correlation between items, validity, and reliability tests.

Step 7. Naming the scale and its sub-items.

Step 8. Confirmatory factor analysis with IBM AMOS.

Step 9. Examining the validity and reliability of the results obtained from confirmatory factor analysis with the IBM AMOS Master Validity (Gaskin, 2023) plug-in.

Step 10. Performing invariance analysis.

The scale will be developed by applying the 10 steps specified.

## 4 Findings

Content validity has an important place in the literature as the minimization of potential errors in the creation of a measurement tool and the support of subsequent stages. Content validity reflects the extent to which the items are related to the measurement tool and their representativeness (Shrotryia and Dhanda, 2019). In this study, the content validity of the scale questions was checked by taking the opinions of 11 experts and presented in Table 2.

**Table 2:** Content validity.

Question	Items	*N*	Ne	CVR	Result
1	I find it difficult to distinguish whether the content I encounter in the digital environment is real or artificial.	11	9	0.6364	Remained
2	The difference between AI-generated content and real content is becoming increasingly blurred.	11	10	0.8182	Remained
3	I feel the need for additional verification to ensure the authenticity of digital content.	11	11	1.0000	Remained
4	I find it difficult to judge how much of the images or videos I see digitally are real.	11	9	0.6364	Remained
5	It is increasingly difficult to trust the accuracy of information presented in the digital environment.	11	9	0.6364	Remained
6	The source of information is not always clear in a digital environment.	11	10	0.8182	Remained
7	It has become difficult for me to assess the reliability of information shared digitally.	11	11	1.0000	Remained
8	Uncertainty about the authenticity of digital content reduces my overall trust in information.	11	10	0.8182	Remained
9	It is often difficult to determine who is responsible for errors in content produced in the digital environment.	11	9	0.6364	Remained
10	It is not clear who is responsible for the data produced by artificial intelligence-supported systems.	11	10	0.8182	Remained
11	Legal accountability for decisions made using digital technologies is becoming increasingly complex.	11	11	1.0000	Remained
12	It is often not possible to identify the source of misinformation in the digital environment.	11	9	0.6364	Remained
13	The ethical boundaries of content that can be produced with digital technologies are not clearly defined.	11	10	0.8182	Remained
14	The ethical implications of many digital applications are not sufficiently questioned.	11	10	0.8182	Remained
15	In digital content production, the difference between “what can be done” and “what should be done” is gradually disappearing.	11	10	0.8182	Remained
16	The weakening of the distinction between the real and the artificial makes ethical evaluations difficult.	11	9	0.6364	Remained
17	Ethical values do not matter in the distinction between the real and the artificial.	11	3	−0.4545	Eliminated

Within the scope of the method proposed by Ayre and Scally (2014), the CVR critical exact values ratio of each item for 11 experts should be at least 0.636. Question 17 in Table 2, which does not meet these conditions, was removed from the question pool. This method ensures that the question pool is developed in accordance with the literature and relevant theories prior to the survey administration and that the scientific quality of the questions is enhanced through expert consultation.

### 4.1 Analysis of the pre-test

Within the scope of the pre-test, Carpenter (2018) states that at least 100 people should be reached. In this context, 162 people were reached, and the participants' demographic information is presented in detail in Table 3.

**Table 3:** Demographic information of pre-test participants.

Items	Group	*n*	%
Gender	Female	48	29.60
	Male	114	70.40
Age	Between the ages of 18 and 30	58	35.80
	Between the ages of 31 and 40	43	26.50
	Between the ages of 41 and 50	38	23.50
	51 years and older	23	14.20
Education status	Bachelor's degree	107	66.00
	Postgraduate	55	34.00
Income	Between 65.001 Turkish Lira and 85.000 Turkish Lira	68	42.00
	Between 85.001 Turkish Lira and 105.000 Turkish Lira	66	40.70
	105,001 Turkish Lira (TL) and above	28	17.30

In Table 3, which includes the demographic characteristics of the teachers and academicians who participated in the study within the scope of the pre-test, it is seen that 70.40% of the participants were male and 29.60% were female. When the age distribution is analyzed, it is noteworthy that the participants are mostly concentrated in the 18–40 age range. This distribution shows that a group that interacts more intensively with digital technologies takes part in the research. In terms of educational level, the fact that all of the participants are higher education graduates indicates that the sample has a high capacity to evaluate digital content. The income distribution shows that the participants are predominantly in the middle- and upper-income groups.

A normality test was conducted to determine the type of analysis (parametric/non-parametric) to be conducted with the collected data, and the results of the normality analysis are presented in Table 4.

**Table 4:** Pre-test normality distribution.

Scale	Kolmogorov-Smirnov			Central tendency measures			
	Statistic	df	Sig.	Mean	Median	Skewness	Kurtosis
Digital blur	0.091	162	0.002	3.687	3.714	−0.403	0.396

When Table 4 is examined, it is determined that Skewness and Kurtosis values are within the range of ± 1.96, and the sample has a normal distribution (Hair et al., 2019). The normality test is of key importance in scale development studies; without ensuring the normality of the data, it is not possible to discuss validity and reliability using parametric analyses. After the normality analysis, correlation analysis was performed to determine the items that did not meet the internal correlation requirement of 0.30 between the items of the scale. The results of the analysis are presented in Table 5.

**Table 5:** Pre-test correlation analysis.

Items	1	2	3	4	5	6	7	8	9	10	11	12	13	14	15	16
Q1	1															
Q2	0.530	1														
Q3	0.580	0.474	1													
Q4	0.649	0.478	0.621	1												
Q5	0.354	0.441	0.474	0.387	1											
Q6	0.288	0.341	0.265	0.237	0.534	1										
Q7	0.412	0.498	0.580	0.527	0.490	0.468	1									
Q8	0.304	0.401	0.350	0.331	0.443	0.486	0.463	1								
Q9	0.236	0.314	0.391	0.356	0.327	0.444	0.468	0.412	1							
Q10	0.364	0.292	0.360	0.367	0.387	0.431	0.402	0.477	0.532	1						
Q11	0.249	0.306	0.315	0.305	0.404	0.403	0.408	0.433	0.496	0.583	1					
Q12	0.254	0.237	0.347	0.345	0.369	0.419	0.333	0.427	0.433	0.477	0.569	1				
Q13	0.286	0.319	0.284	0.242	0.318	0.406	0.391	0.422	0.486	0.529	0.596	0.497	1			
Q14	0.253	0.324	0.295	0.210	0.508	0.483	0.456	0.426	0.394	0.435	0.535	0.490	0.565	1		
Q15	0.322	0.471	0.333	0.234	0.446	0.558	0.475	0.539	0.458	0.501	0.623	0.511	0.632	0.662	1	
Q16	0.307	0.350	0.306	0.284	0.492	0.457	0.401	0.455	0.418	0.470	0.528	0.555	0.568	0.568	0.634	1

All values in Table 5 are significant at the 0.01 level. When Table 5 is examined, it is seen that each item has a relationship of 0.30 with at least one other item (Hair et al., 2019). Therefore, it was determined that there was no need to remove any question from the scale. The results obtained show that the data are suitable for exploratory factor analysis (EFA). The exploratory factor analysis is presented in Table 6.

**Table 6:** Pre-test exploratory factor analysis.

Items	Factor load value (SPSS)	Cronbach's alpha (α)	PA results		
			(Ncases: 162; Nvar: 16; Ndataset:100; Percent: 95; Brian Oc)		
			Raw data	Means	Percently
**Information and ethical blur**		α = 0.908	6.502	1.584	1.704
% of variance: 43.058; eigen-value: 6.502					
Question 6	0.618				
Question 8	0.553				
Question 9	0.539				
Question 10	0.593				
Question 11	0.765				
Question 12	0.643				
Question 13	0.789				
Question 14	0.795				
Question 15	0.880				
Question 16	0.750				
**Reality blur**		α = 0.833	1.734	1.449	1.526
% of variance: 9.489; eigen-value: 1.734					
Question 1	0.781				
Question 2	0.507				
Question 3	0.714				
Question 4	0.880				
Extraction method: maximum likelihood (ML)					
Rotation method: direct oblimin					
KMO: 0.922;					
Bartlett's sphericity test; (χ2 = 1,110.628; df = 91; p = 0.000)					
Cronbach's alpha (α) = 0.910					

The values in bold in the table indicate the reliability of the analysis.

Exploratory factor analysis was conducted using the Maximum Likelihood (ML) method and Direct Oblimin rotation as suggested by Carpenter (2018). In the first analysis, Question 5 and Question 7 items were removed from the scale because they did not obtain sufficient factor loading values of 0.50. Applying this threshold for factor loadings is related to ensuring the adequacy of internal validity values. When the analysis was repeated, all items received values above 0.50, and the scale items were distributed in a binary factor structure (Hair et al., 2019). The two newly formed factors were named Information and Ethical Blurring (IE Blurring) and Reality Blurring in accordance with the literature. The naming of the scales is based on the fact that they contain information derived from the fundamentals of SIP Theory, their alignment with ethical values, and their reference to reality. The names given to the scale dimensions are consistent with the content of the current questions (Marmat, 2022). Since the Kaiser-Meyer-Olkin sampling adequacy test result of the exploratory factor analysis was above 0.70, the *p*-value of Bartlett's test of sphericity was less than 0.05, and the explained variance value was 52.547%, which is higher than 50%, the test was found to be significant. In the validity and reliability analysis conducted for the scale, the Cronbach's Alpha coefficient of the Information and Ethical Blurring sub-dimension was 0.908, the Cronbach's Alpha coefficient of the Reality Blurring sub-dimension was 0.833, and the Cronbach's Alpha coefficient of the whole scale was 0.910. A Cronbach's Alpha coefficient above 0.70 indicates that sufficient validity and reliability are achieved (Hair et al., 2019). As a result of the analysis conducted with the parallel analysis values indicated in the upper right corner of Table 5, the two sub-dimensional structures of the scale were confirmed. In addition, the Kaiser-Guttman Criteria test was conducted to check the sub-dimensionality of the scale. All of the analyses conducted at this stage are intended to demonstrate the statistical appropriateness, validity, and reliability of the scale structure defined based on the literature and theory.

In the Kaiser-Gutman Criteria test analysis presented in Table 7, eigenvalues greater than one are used to determine the number of dimensions (Hair et al., 2019). In this context, it is seen that it is proven again that the scale has two sub-dimensions.

**Table 7:** Unidimensionality analysis (Kaiser-Gutman criteria).

Scale	Items number	1. Eigenvalue	2. Eigenvalue	3. Eigenvalue	4. Eigenvalue	Total variance
Digital blur	14	7.388	1.806	0.944	0.761	57.461

**Figure 1:**
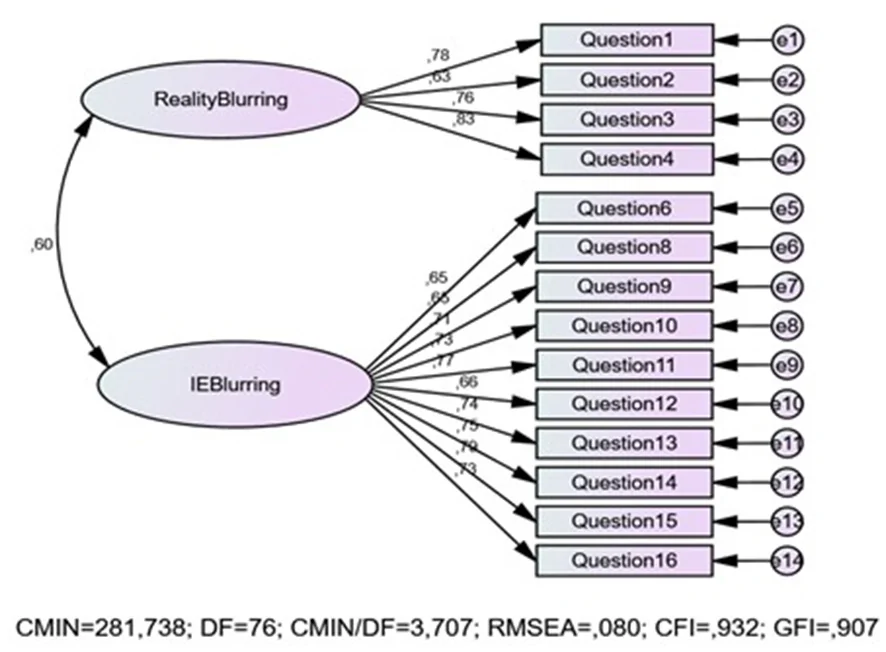
Pretest path diagram.

In previous analyses, the scale's structure, validity, and reliability values were determined using SPSS. According to the literature, to verify the accuracy of the results obtained, a more complex and valid confirmatory factor analysis must be conducted using AMOS. Confirmatory factor analysis (CFA) provides information about the statistical significance of scales by examining their observed measurements and latent variable structure. In this context, various values stand out. Some of these include a X^2^(df) value less than 5, a *p*-value less than 0.05, Root Mean Square Error of Approximation (RMSEA) and Standardized Root Mean Square Residual (SRMR) values less than 0.080, a Goodness-of-Fit Index (GFI) value, and Comparative Fit Index (CFI), and a Normalized Fit Index (CFI) value greater than 0.90 are interpreted as indicators of good fit (Hair et al., 2019). The results obtained through CFA demonstrate that the scale possesses adequate statistical values, and the path diagram of the analysis is presented in Figure 1.

The visuals of the path diagram were taken directly from the AMOS program to show that the analysis was actually performed and to prove that the values were provided. However, since this application technically prevents the sharing of some values, these missing values are presented in Table 8.

**Table 8:** Pretest goodness of fit values.

X^2^(df)	*p*	RMSEA	CFI	GFI	NFI	SRMR
1.441	0.007	0.052	0.968	0.903	0.905	0.053

An examination of Table 8 reveals that all the obtained values fall within the range of goodness-of-fit values. The results indicate that the structure of the scale, as determined by the analysis conducted using SPSS software, has been validated using AMOS software. The measurement model showing the details of the analysis is presented in Table 9.

**Table 9:** Pre-test measurement model.

Measurement model			β1	β2	Ss	*t*	*p*
Question 1	< –	Reality Blurring	0.791	1.000			
Question 2	< –	Reality Blurring	0.650	0.708	0.088	8.022	< 0.001
Question 3	< –	Reality Blurring	0.757	0.957	0.102	9.422	< 0.001
Question 4	< –	Reality Blurring	0.795	0.942	0.096	9.850	< 0.001
Question 6	< –	IE Blurring	0.631	1.000			
Question 8	< –	IE Blurring	0.634	1.043	0.149	6.981	< 0.001
Question 9	< –	IE Blurring	0.627	0.921	0.133	6.914	< 0.001
Question 10	< –	IE Blurring	0.689	1.026	0.138	7.461	< 0.001
Question 11	< –	IE Blurring	0.756	1.100	0.137	8.019	< 0.001
Question 12	< –	IE Blurring	0.684	1.175	0.158	7.424	< 0.001
Question 13	< –	IE Blurring	0.749	1.183	0.149	7.964	< 0.001
Question 14	< –	IE Blurring	0.728	1.121	0.144	7.793	< 0.001
Question 15	< –	IE Blurring	0.822	1.121	0.131	8.530	< 0.001
Question 16	< –	IE Blurring	0.741	1.211	0.153	7.902	< 0.001
Reality blurring	< ->	IE Blurring	0.552	0.310	0.066	4.671	< 0.001

Upon examining the measurement model, a correlation coefficient of 0.552 is observed between the two subscales. The fact that this value is above 0.50 indicates a sufficient and meaningful relationship between the scale's subscales. In short, the obtained value demonstrates that the scales are interconnected and related to one another. Furthermore, the fact that the item factor loadings used to calculate the validity and reliability values in CFA are higher than 0.50 is a positive result. Factor loadings serve as the primary inputs in the calculation of the Combined Reliability (CR) and Average Variance Extracted (AVE) values, which indicate validity and reliability (Hair et al., 2019).

In confirmatory factor analysis, combined reliability (CR) is used to measure the overall reliability level of multiple, heterogeneous, but similar statements. Average Variance Extracted (AVE) value is an indicator obtained by dividing the sum of the squares of the item factor loadings for the scale or sub-dimension by the number of statements. For reliability and validity, the CR value should be above 0.70, and the AVE value should be above 0.50. Maximum Squared Variance (MSV) refers to the square of the maximum variance that a factor shares with any of the other factors. Maximum Reliability (MaxR(H)) is an indicator that assesses the measurement power of a construct or latent variable (Yaşlioglu, 2017). When the CR value is higher than the AVE value, convergent validity is ensured; when the AVE value is greater than the MSV value, divergent validity is ensured; and when the MaxR(H) value is higher than the CR value, construct validity is ensured (Hair et al., 2019). Related values are presented in Table 10.

**Table 10:** Pretest validity, reliability and discriminant validity.

Items	CR	AVE	MSV	MaxR(H)	Reality blur	IE blur
Reality blur	0.837	0.563	0.305	0.845	0.751^*^	0.588^**^
IE blur	0.909	0.502	0.305	0.915	0.552^*^	0.709^*^

^*^Fornell and Lacker Criteria, ^**^HTMT Ratio.

When Table 10 is examined, it is seen that divergent, convergent, and construct validity of the scale are provided. At the same time, it was determined that the discriminant validity, which shows that the items of the scale are separated from each other, was also provided within the scope of Fornell and Larcker Criterion and HTMT values (Fornell and Larcker, 1981; Henseler et al., 2015).

The results of the pre-test indicate that the scale's structure, validity, and reliability values, and EFA and CFA analyses fall within the ranges specified in the literature. The results of all the procedures conducted demonstrate that the Digital Blur Scale, developed in accordance with the literature and SIP theory, is statistically significant and suitable for use. To confirm that the scale yields the same results across different time periods and with different samples, it must be tested with a larger sample. In this context, the scale will be retested using the main sample.

### 4.2 Analysis of the main test

Unlike the pretest, in the second analysis, the sample was expanded to include the general population and reached a heterogeneous structure. The demographic data of the participants included in the second analysis are presented in Table 11 below.

**Table 11:** Demographic variables.

Items	Group	*n*	%
Gender	Female	199	47.30
	Male	222	52.70
Age	Between the ages of 18 and 22	169	40.10
	Between the ages of 23 and 27	45	10.70
	Between the ages of 28 and 32	25	6.00
	Between the ages of 33 and 37	38	9.00
	Between the ages of 38 and 42	47	11.20
	Between the ages of 43 and 47	48	11.40
	Between the ages of 48 and 52	26	6.20
	53 years and older	23	5.40
Education status	High school and below	32	7.60
	Associate's degree	72	17.10
	Bachelor's degree	211	50.10
	Postgraduate	106	25.20
Occupation	Civil servant	85	20.20
	Student	223	53.00
	Academician	66	15.60
	Private sector employee	26	6.20
	Shopkeeper	21	5.00
Income	25.000 TL and below	199	47.30
	Between 25.001 TL and 45.000 TL	37	8.80
	Between 45.001 TL and 65.000 TL	24	5.70
	Between 65.001 TL and 85.000 TL	48	11.40
	Between 85.001 TL and 105.000 TL	49	11.60
	105.001 TL and above	64	15.20

Table 11 shows that the gender distribution is balanced (52.70% male; 47.30% female). This shows that a more balanced sample structure was reached compared to the first phase. When the age distribution is analysed, it is seen that the participants are significantly concentrated in the younger groups, and especially the 18–22 age group (40.10%) has a significant weight in the sample. However, the inclusion of older age groups in the sample shows that a wider distribution in terms of age has been achieved. In terms of educational level, it is seen that the majority of the participants are bachelor's (50.10%) and postgraduate (25.20%) graduates, although high school and associate degree graduates are also included in the sample. In terms of occupational distribution, more than half of the participants were students (53.00%), while civil servants (20.20%), academics (15.60%) and other occupational groups were also included in the sample. This diversity shows that the research is not limited to a specific occupational group but reaches a wider audience. The income distribution reveals that the participants represent different socioeconomic levels. In the second phase of the research, the sample structure was expanded and a participant profile including more diverse demographic characteristics was reached. This increases the generalizability of the findings and allows for a more comprehensive examination of the reflections of the concept of digital blur on different individual and socioeconomic groups.

The normality analysis results of the data collected from this sample are presented in Table 12.

**Table 12:** Normality distribution.

Scale	Kolmogorov-Smirnov			Central tendency measures			
	Statistic	df	Sig.	Mean	Median	Skewness	Kurtosis
Digital blur	0.080	421	0.000	3.669	3.714	−0.734	1.473

When Table 12 is examined, it is determined that Skewness and Kurtosis values are within the range of ± 1.96 and the sample is normally distributed. After the normality analysis, correlation analysis was performed to determine the item that did not meet the internal correlation requirement of 0.30 between the items of the scale (Hair et al., 2019). The results of the analysis are presented in Table 13.

**Table 13:** Correlation analysis.

Items	1	2	3	4	5	6	7	8	9	10	11	12	13	14
1	1													
2	0.470	1												
3	0.582	0.486	1											
4	0.685	0.485	0.630	1										
6	0.287	0.396	0.336	0.300	1									
8	0.341	0.411	0.366	0.418	0.542	1								
9	0.269	0.383	0.393	0.362	0.505	0.458	1							
10	0.329	0.327	0.389	0.366	0.445	0.493	0.618	1						
11	0.273	0.391	0.390	0.347	0.451	0.472	0.587	0.635	1					
12	0.258	0.284	0.301	0.349	0.406	0.411	0.475	0.512	0.521	1				
13	0.278	0.393	0.324	0.295	0.437	0.402	0.521	0.501	0.575	0.523	1			
14	0.256	0.375	0.322	0.276	0.489	0.420	0.482	0.506	0.550	0.479	0.638	1		
15	0.311	0.458	0.405	0.341	0.505	0.529	0.462	0.547	0.629	0.480	0.584	0.676	1	
16	0.296	0.437	0.351	0.323	0.520	0.474	0.501	0.467	0.502	0.486	0.549	0.579	0.621	1

All values in Table 13 are significant at the 0.01 level. When Table 13 is examined, it is seen that each item has a relationship of 0.30 with at least one other item (Hair et al., 2019). Therefore, it was determined that there was no need to remove any question from the scale. The results obtained show that the data are suitable for exploratory factor analysis. The exploratory factor analysis is presented in Table 14.

**Table 14:** Exploratory factor analysis.

Items	Factor load value (SPSS)	Cronbach's alpha (α)	PA results		
			(Ncases: 421; Nvar: 14; Ndataset:100; Percent: 95; Brian Oc)		
			Raw data	Means	Percently
**Information and ethical blur**		α = 0.914	6.791	1.319	1.384
% of variance: 45.079; eigen-value: 6.791					
Question6	0.649				
Question8	0.635				
Question9	0.700				
Question10	0.721				
Question11	0.768				
Question12	0.652				
Question13	0.745				
Question14	0.766				
Question15	0.800				
Question16	0.731				
**Reality blur**		α = 0.834	1.607	1.247	1.297
% of variance: 8.841; eigen-value: 1.607					
Question1	0.794				
Question2	0.593				
Question3	0.741				
Question4	0.857				
Extraction method: maximum likelihood (ML)					
Rotation method: direct oblimin					
KMO: 0.930;					
Bartlett's sphericity test; (χ2 = 3,089.565; df = 91; p = 0.000)					
Cronbach's alpha (α) = 0.917					

The values in bold in the table indicate the reliability of the analysis.

Exploratory factor analysis was conducted using the Maximum Likelihood (ML) method proposed by Carpenter (2018) and Direct Oblimin rotation. All items in the analysis were distributed to the scale in a binary factor structure with a value above 0.50. It was determined that there was no difference between the new analysis and the pre-test analysis. Since the Kaiser-Meyer-Olkin sampling adequacy test result of the exploratory factor analysis was above 0.70, the *p*-value of Bartlett's test of sphericity was less than 0.05, and the explained variance value was 53.920%, which is higher than 50%, the test was significant. In the validity and reliability analysis conducted for the scale, the Cronbach's Alpha coefficient of the Information and Ethical Blurring sub-dimension was 0.914, the Cronbach's Alpha coefficient of the Reality Blurring sub-dimension was 0.834, and the Cronbach's Alpha coefficient of the whole scale was 0.917. A Cronbach's Alpha coefficient above 0.70 indicates that sufficient validity and reliability are achieved (Hair et al., 2019). As a result of the analysis conducted with the parallel analysis values indicated in the upper right corner of Table 13, the two sub-dimensional structures of the scale were confirmed. In addition, the Kaiser-Guttman Criteria test was conducted to check the sub-dimensionality of the scale. This test is presented in Table 15.

**Table 15:** Unidimensionality analysis (Kaiser-Gutman criteria).

Scale	Items number	1. Eigen value	2. Eigen value	3. Eigen value	4. Eigen value	Total variance
Digital blur	14	6.791	1.607	0.787	0.731	59.983

**Figure 2:**
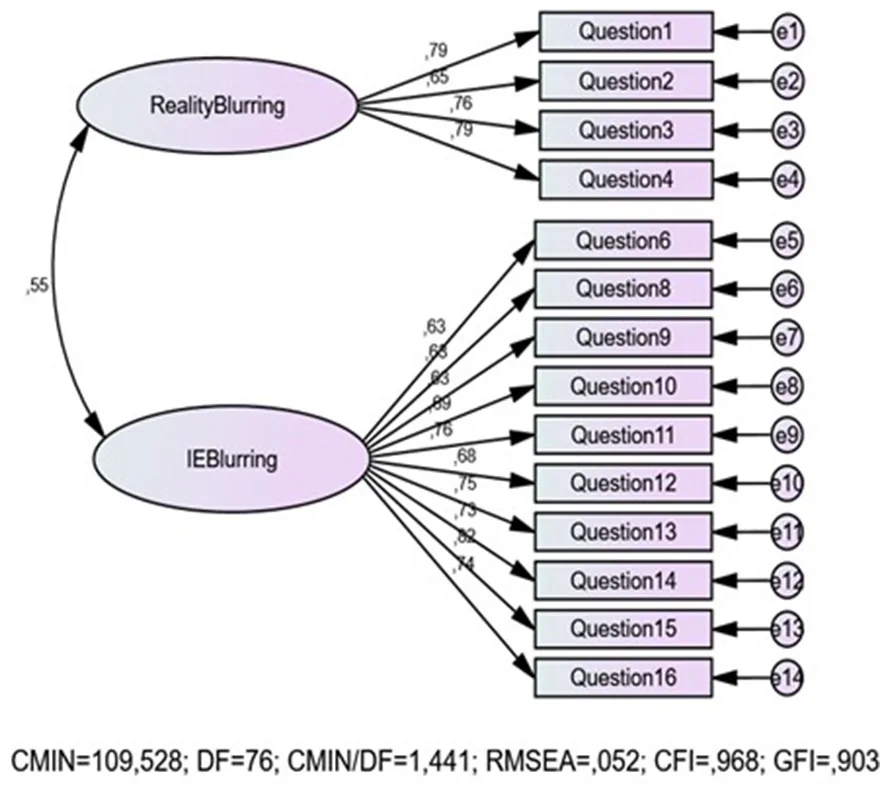
Main test path diagram.

Eigenvalues greater than 1 in Table 15 are used to determine the number of dimensions (Hair et al., 2019). In this context, it is seen that it is proven again that the scale has two sub-dimensions. With the results obtained, it was determined that the scale had sufficient statistical values for confirmatory factor analysis, and the path diagram of the analysis is presented in Figure 2.

As it can be seen from Figure 2, it is seen that goodness of fit values are provided as a result of CFA analysis and detailed information is presented in Table 16.

**Table 16:** Goodness of fit values.

X^2^(df)	*p*	RMSEA	CFI	GFI	NFI	SRMR
3.707	0.007	0.080	0.932	0.907	0.910	0.053

When Table 16 is examined, it is seen that all of the values obtained are within the goodness-of-fit values. The measurement model showing the details of the analysis is presented in Table 17.

**Table 17:** Measurement model.

Measurement model			β1	β2	Ss	*t*	*p*
Question 1	< –	Reality Blurring	0.779	1.000			
Question 2	< –	Reality Blurring	0.629	0.665	0.053	12.525	< 0.001
Question 3	< –	Reality Blurring	0.764	0.951	0.062	15.434	< 0.001
Question 4	< –	Reality Blurring	0.830	0.990	0.060	16.594	< 0.001
Question 6	< –	IE Blurring	0.653	1.000			
Question 8	< –	IE Blurring	0.645	1.040	0.088	11.792	< 0.001
Question 9	< –	IE Blurring	0.707	1.097	0.086	12.746	< 0.001
Question 10	< –	IE Blurring	0.732	1.124	0.086	13.118	< 0.001
Question 11	< –	IE Blurring	0.770	1.160	0.085	13.674	< 0.001
Question 12	< –	IE Blurring	0.658	1.046	0.087	11.990	< 0.001
Question 13	< –	IE Blurring	0.737	1.178	0.089	13.191	< 0.001
Question 14	< –	IE Blurring	0.754	1.129	0.084	13.439	< 0.001
Question 15	< –	IE Blurring	0.793	1.084	0.077	14.002	< 0.001
Question 16	< –	IE Blurring	0.728	1.125	0.086	13.058	< 0.001
Reality Blurring	< ->	IE Blurring	0.599	0.336	0.042	7.995	< 0.001

When the measurement model is examined, it is seen that there is a correlational value of 0.599 between the two sub-dimensions. The fact that this value is above 0.50 indicates that there is a sufficient and significant relationship between the sub-dimensions of the scale (Hair et al., 2019). In addition, it is a positive result that the factor loading values of the scale items, which is an indicator of validity and reliability, are higher than 0.50. The analyses mentioned so far do not provide information about the validity and reliability of the results obtained. Therefore, new calculations are required. The relevant values are presented in Table 18.

**Table 18:** Validity, reliability and discriminant validity.

Items	CR	AVE	MSV	MaxR(H)	Reality blur	IE blur
Reality blur	0.839	0.569	0.359	0.853	0.754^*^	0.640^**^
IE blur	0.914	0.517	0.359	0.918	0.599^*^	0.719^*^

^*^Fornell and Lacker Criteria, ^**^HTMT Ratio.

When Table 18 is examined, it is seen that divergent, convergent and construct validity of the scale is provided. At the same time, it was determined that the discriminant validity, which shows that the items of the scale are separated from each other, was also provided within the scope of Fornell and Lacker Criterion and HTMT values (Fornell and Larcker, 1981; Henseler et al., 2015).

Confirmatory factor analysis (CFA) is used for psychometric evaluation and construct validation of scales. In addition, this method is also used to evaluate factor invariance by comparing the results obtained with more than one analysis (Byrne, 2016). In this context, 162 and 421 samples will be subjected to invariance analysis through AMOS program. The obtained result reveals the ability of the scale to give similar results in different samples. As suggested by Byrne (2016) for the analysis, CFI values will be checked through multiple group analysis. The values for the invariance analysis are presented in Table 19.

**Table 19:** Invariance analysis.

Model	χ^2^	df	χ^2^/df	NFI	CFI	RMSEA	*Δχ* ^2^	Δdf	ΔCFI	*p*-value for *Δχ*^2^
Group 1	109.528	76	1.441	0.905	0.968	0.052	–	–	–	
Group 2	281.738	76	3.707	0.910	0.932	0.080	–	–	–	
Model 1: configural	391.271	152	2.574	0.909	0.942	0.052	–	–	–	
Model 2: weak (metric)	397.474	164	2.424	0.907	0.943	0.05	6.203	12	0.001	0.906
Model 3: scalar	402.525	178	2.261	0.906	0.945	0.047	5.051	14	0.002	0.985
Model 4: strong	404.016	181	2.232	0.906	0.946	0.046	1.491	3	0.001	0.684
Model 5: partial	412.038	195	2.113	0.904	0.947	0.044	8.022	14	0.001	0.888

Δχ^2^: χ^2^ change (|$\chi_{\text{n}}^{2}$-$\chi_{\text{n}-1}^{2}$|); Δdf: df change (|df_n_-df_n − 1_|); Δχ^2^/df: χ^2^/df change (|$\chi_{\text{n}}^{2}$/df_n_-| $\chi_{\text{n}-1}^{2}$/df_n − 1_); ΔCFI: CFI change (|CFI_n_-CFI_n − 1_|); ΔCFI < 0.01; *p*-value for Δχ^2^: χ^2^ *significance value of change* (*p* < 0.05^*^).

When Table 19 is examined, it is determined that the scale meets the invariance conditions as the ΔCFI value is below 0.01 (Cheung and Rensvold, 2002) in the comparison between both samples. Therefore, it is possible to state that the scale has invariance and is suitable to be used by large masses.

The results of the pre-test and main test, along with the scale's structure, validity, and reliability values, indicate that the EFA and CFA analyses fall within the ranges specified in the literature. Combined with the invariance analysis, it has been demonstrated that the scale yields the same results across different time periods and with different samples. The statistical significance of the Digital Blur Scale provides strong evidence of its alignment with the literature and SIP theory.

## 5 Discussion

### 5.1 Practical implications

This study makes three main contributions to the related literature. First, it proposes a new conceptual framework that explains the transformation of reality perception in the digital age. In the existing literature, problems related to digital environments are mostly addressed within the framework of misinformation and disinformation (Lewandowsky et al., 2017; Wardle and Derakhshan, 2017) or discussed in terms of the transparency and technical structures of algorithmic systems (Burrell, 2016). However, these approaches are insufficient to explain why and how the reality status of digital content, regardless of its accuracy, cannot be assessed by individuals. Through the concept of digital blur, this study offers a holistic explanation centering on the perceptual and epistemic weakening of the distinction between the real and the artificial, and in this respect, it provides a new conceptual ground for understanding the erosion of trust in data in the digital age. This finding supports the argument that information in the digital environment can not only be inaccurate but also epistemically ambiguous (Floridi, 2014).

The second contribution is the development of a psychometrically valid and reliable instrument to measure this concept. Although there are various scales related to digital environments in the literature, most of these scales focus on cognitive overload processes such as digital overload or technostress (Tarafdar et al., 2015; La Torre et al., 2019). However, there is no tool that directly measures uncertainty about the reality status of digital content. The scale developed within the scope of this study was subjected to multidimensional tests such as content validity, exploratory and confirmatory factor analyses, parallel analysis and discriminant validity and yielded strong statistical results. The two-stage sampling structure of the study was an important factor that increased the reliability and generalizability of the findings. In the first stage, the scale was developed with a more homogeneous sample of teachers and academics, and in the second stage, it was tested with a larger and heterogeneous group of participants. The consistency of the findings obtained in the two stages reveals that the developed measurement tool is valid for different demographic groups. These results reveal that digital blur is an empirically measurable construct and fill an important methodological gap in the literature.

The third contribution of the research is to provide a new analytical perspective for understanding individual and institutional processes of evaluating digital content. At this point, the findings show that uncertainty in digital environments is experienced by individuals not in the form of differentiated dimensions, but as a more holistic epistemic uncertainty. The fact that the theoretically constructed multidimensional structure exhibits a more integrated structure in empirical analyses reveals that individuals consider information accuracy, trust, responsibility and ethics as intertwined rather than independent of each other when evaluating digital content. This is in line with the findings in the literature on how trust is formed especially in digital environments (van der Linden et al., 2020). At the same time, this result is in line with studies showing that synthetic media and deepfake technologies lead to a general state of uncertainty and skepticism in individuals, not only misinformation (Chesney and Citron, 2019; Vaccari and Chadwick, 2020).

### 5.2 Theoretical implications

SIP Theory is an important theory used to explain how people process information in computer-mediated communication. Over time, the theory has gained widespread acceptance and has been adopted to explain its effects in many areas, including social media communication and relationships (Walther, 1992). With today's advancing technology, it has been shown that content generated by artificial intelligence causes erosion in people's perceptions of reality, leading to a sense of blurring. This study explains this uncertainty using SIP Theory, thereby making a small but significant contribution to the expansion of the theory. The study's findings demonstrate that the SIP Theory is effective in describing the uncertainty individuals experience in the digital realm. From this perspective, the study shows that in digital environments, where information is reproduced by artificial intelligence, people's perception of reality is undergoing erosion, and their information processing becomes increasingly complex (Marmat, 2022).

When the findings are evaluated in a broader theoretical context, on the basis of Social Information Processing Theory, it can be argued that digital blur is one of the main mechanisms contributing to the systematic erosion of epistemic trust in the digital age. In particular, the uncertainty that individuals experience in the evaluation processes of digital content points to a deeper deterministic transformation that cannot be explained solely by exposure to misinformation. This situation may cause individuals to develop suspicion toward correct information and create a general erosion of trust (Lewandowsky et al., 2017). Therefore, digital blur can be considered as a structural problem area that requires rethinking the processes of evaluating information in the digital environment.

### 5.3 Strengths, limitations, and future research

Within the framework of all these results, this study reveals that the perception of reality in the digital age is not only weakening but also structurally transforming, and that the concept of digital blur, which was developed to explain this transformation, provides a powerful framework both theoretically and empirically. Digital blur can be considered as a fundamental conceptual and analytical tool for understanding and analysing the epistemic uncertainty emerging in the digital information ecosystem. In addition, as in every academic research, this study has some limitations. First of all, since the research data were collected through self-report method, there may be a risk of bias based on participants' perceptions. In addition, the fact that the sample is focused on certain groups, especially students and young participants, may limit the generalizability of the findings. Again, since the study had a cross-sectional design, it was not possible to evaluate the change in the concept of digital blur over time and causal relationships. Therefore, it is recommended that future studies should be supported with different sample groups and longitudinal designs.

## 6 Conclusion

In this study, the concept of “digital blur” was proposed in order to explain the uncertainty of reality that has become increasingly evident in the digital age, and a valid and reliable scale was developed to measure this concept. The findings of the study show that not only the accuracy but also the reality status of information in digital environments cannot be reliably evaluated by individuals and this situation creates a systematic area of uncertainty. The analyses conducted within the scope of the study revealed that the developed scale had high validity and reliability values and was consistent across different samples. This shows that the concept of digital blur is not only a theoretical proposal, but also an empirically measurable construct.

## Data Availability

The original contributions presented in the study are included in the article/Supplementary material, further inquiries can be directed to the corresponding author.

## References

[B1] Abdulkareem A. K. Ishola A. A. Bello M. L. Adejumo A. (2024). The dark side of digitalization: examining the impact of digital overload on job autonomy and job satisfaction. J. Inf. Commun. Ethics Soci. 22, 354–371. doi: 10.1108/JICES-07-2023-0091

[B2] Adams Z. Osman M. Bechlivanidis C. Meder B. (2023). (Why) is misinformation a problem? Perspect. Psychol. Sci. 18, 1436–1463. doi: 10.1177/17456916221141344 36795592 PMC10623619

[B3] Alatoai A. A. Alshahri A. S. (2025). The development and validation of the AI-supported self-regulated science and mathematics learning scale (AI-SSRSML) among secondary school students in Saudi Arabia. BMC Psychol. 13:1330. doi: 10.1186/s40359-025-03764-z 41299789 PMC12670807

[B4] Alsabah M. Naser M. A. Albahri A. S. Albahri O. S. Alamoodi A. H. Abdulhussain S. H. . (2025). A comprehensive review on key technologies toward smart healthcare systems based IoT: technical aspects, challenges and future directions. Artif. Intell. Rev. 58*:*343. doi: 10.1007/s10462-025-11342-3

[B5] Ayre C. Scally A. J. (2014). Critical values for Lawshe's content validity ratio: revisiting the original methods of calculation. Meas. Eval. Couns. Dev. 47, 79–86. doi: 10.1177/0748175613513808

[B6] Bilişli Y. Çakmak F. Zetter S. A. Ünal M. I. (2024). Navigating truth and disinformation: a comparative analysis of generational responses to the 6 February 2023 earthquake in digital media in Türkiye. Heliyon 10:e38667. doi: 10.1016/j.heliyon.2024.e38667 39397969 PMC11471220

[B7] Burrell J. (2016). How the machine ‘thinks': understanding opacity in machine learning algorithms. Big Data Soc. 3, 1–12. doi: 10.1177/2053951715622512

[B8] Byrne B. M. (2016). Structural Equation Modeling with Amos: Basic Concepts, Applications, and Programming. New York, NY: Taylor and Francis. doi: 10.4324/9781315757421

[B9] Carpenter S. (2018). Ten steps in scale development and reporting: a guide for researchers. Commun. Methods Meas. 12, 25–44. doi: 10.1080/19312458.2017.1396583

[B10] Chesney B. Citron D. (2019). Deep fakes: a looming challenge for privacy, democracy, and national security. Calif. Law Rev. 107, 1753–1820. doi: 10.2139/ssrn.3213954

[B11] Cheung G. W. Rensvold R. B. (2002). Evaluating goodness-of-fit indexes for testing measurement invariance. Struct. Equ. Model. 9, 233–255. doi: 10.1207/S15328007SEM0902_5

[B12] Citron D. K. (2019). Sexual privacy. Yale Law J. 128, 1870–1960. Available online at: https://scholarship.law.bu.edu/faculty_scholarship/620

[B13] Citron D. K. Pasquale F. (2014). The scored society: due process for automated predictions. Wash. Law Rev. 89, 1–33. Available online at: https://digitalcommons.law.uw.edu/wlr/vol89/iss1/2

[B14] Cochran W. G. (1977). Sampling Techniques. New York, NY: Wiley.

[B15] Feliciano-Cestero M. M. Ameen N. Kotabe M. Paul J. Signoret M. (2023). Is digital transformation threatened? A systematic literature review of the factors influencing firms' digital transformation and internationalization. J. Bus. Res. 157:113546. doi: 10.1016/j.jbusres.2022.113546

[B16] Floridi L. (2014). The 4th. Revolution. Oxford: Oxford Press, 248.

[B17] Floridi L. Cowls J. Beltrametti M. Chatila R. Chazerand P. Dignum V. . (2018). AI4people-an ethical framework for a good AI society: opportunities, risks, principles, and recommendations. Minds Mach. 28, 689–707. doi: 10.1007/s11023-018-9482-5 30930541 PMC6404626

[B18] Fornell C. Larcker D. F. (1981). Evaluating structural equation models with unobservable variables and measurement error. J. Market. Res. 18:39. doi: 10.1177/002224378101800313

[B19] García-Navarro C. Pulido-Martos M. (2026). Erudit AI SaaS: an artificial intelligence tool based on RoBERTa for classifying employee engagement. BMC Psychol. 14:703. doi: 10.1186/s40359-026-04383-y 41928345 PMC13169601

[B20] Gaskin J. E. (2023). Plugins - Gaskination StatWiki. Available online at: https://statwiki.gaskination.com/index.php/Plugins (accessed March 11, 2026).

[B21] Guo C. Liu H. Song F. Guo J. (2025). The double-edged sword effects of algorithmic opacity: the self-determination theory perspective. Acta Psychol. 260:105600. doi: 10.1016/j.actpsy.2025.105600 40997447

[B22] Habermas J. (2006). Political communication in media society: does democracy still enjoy an epistemic dimension? The impact of normative theory on empirical research. Commun. Theory 16, 411–426. doi: 10.1111/j.1468-2885.2006.00280.x

[B23] Hair J. F. Babin B. J. Black W. C. Anderson R. E. (2019). Multivariate Data Analysis. Hampshire: Cengage Learning.

[B24] Henseler J. Ringle C. M. Sarstedt M. (2015). A new criterion for assessing discriminant validity in variance-based structural equation modeling. J. Acad. Mark. Sci. 43, 115–135. doi: 10.1007/s11747-014-0403-8

[B25] Hynek N. Gavurova B. Kubak M. (2025). Risks and benefits of artificial intelligence deepfakes: systematic review and comparison of public attitudes in seven European Countries. J. Innov. Knowl. 10:100782. doi: 10.1016/j.jik.2025.100782

[B26] Kellogg K. C. Valentine M. A. Christin A. (2020). Algorithms at work: the new contested terrain of control. Acad Manag Ann. 14, 366–410. doi: 10.5465/annals.2018.0174

[B27] Khurana P. Krishnan A. (2025). Communicating through #hashtags: influencing perceptions of personality and trust. Comput. Human Behav. 168:108623. doi: 10.1016/j.chb.2025.108623

[B28] Kim Y. J. Zhang C. X. Fang C. (2025). Navigating the human-AI divide: boundary work in the age of generative AI. Comput. Hum. Behav. 6:100214. doi: 10.1016/j.chbah.2025.100214

[B29] Koçbaş Ö. A. (2025). Hakikat Sonrasi Çagda Sentetik Medya Olgusu: Gerçeklik Algisi ve Etik Sorunsali. 4. BOYUT Med. ve Kült. Çaliş. Derg. 0, 124–145. doi: 10.26650/4boyut.2025.1623917

[B30] La Torre G. De Leonardis V. Chiappetta M. (2020). Technostress: how does it affect the productivity and life of an individual? Results of an observational study. Public Health 189, 60–65. doi: 10.1016/j.puhe.2020.09.013 33166856

[B31] La Torre G. Esposito A. Sciarra I. Chiappetta M. (2019). Definition, symptoms and risk of techno-stress: a systematic review. Int. Arch. Occup. Environ. Health 92, 13–35. doi: 10.1007/s00420-018-1352-1 30196317

[B32] Lee D. Wang L. Lee F. Song Z. Lee D. Wang L. . (2026). Government digital initiatives and enterprise digital transformation: roles of TMTs attention allocation and resource orchestration capability. J. East Eur. Manage. Stud. 31:39933. doi: 10.31083/JEEMS39933

[B33] Lewandowsky S. Ecker U. K. H. Cook J. (2017). Beyond misinformation: understanding and coping with the “post-truth” era. J. Appl. Res. Mem. Cogn. 6, 353–369. doi: 10.1016/j.jarmac.2017.07.008

[B34] Liu X. Li Y. (2025). Examining the double-edged sword effect of AI usage on work engagement: the moderating role of core task characteristics substitution. Behav. Sci. 15:206. doi: 10.3390/bs15020206 40001838 PMC11852299

[B35] Loh J. Walsh M. J. (2021). Social media context collapse: the consequential differences between context collusion versus context collision. Soc. Media Soc. 7, 1–15. doi: 10.1177/20563051211041646

[B36] Marmat G. (2022). Online brand communication and building brand trust: social information processing theory perspective. Glob. Knowl. Mem. Commun. 71, 584–604. doi: 10.1108/GKMC-12-2020-0195

[B37] Marwick A. E. Boyd D. (2011). I tweet honestly, I tweet passionately: twitter users, context collapse, and the imagined audience. New Media Soc. 13, 114–133. doi: 10.1177/1461444810365313

[B38] Massa S. Annosi M. C. Marchegiani L. Messeni Petruzzelli A. (2023). Digital technologies and knowledge processes: new emerging strategies in international business. A systematic literature review. J. Knowl. Manage. 27, 330–387. doi: 10.1108/JKM-12-2022-0993

[B39] Mortazavi Ravari S. S. Mehrabanfar E. Banaitis A. Banaitiene N. (2016). Framework for assessing technological innovation capability in research and technology organizations. J. Bus. Econom. Manage. 17, 825–847. doi: 10.3846/16111699.2016.1253607

[B40] O'Connor B. (2000). Parallel Analysis. Available online at: https://global.oup.com/us/companion.websites/9780199734177/supplementary/factors/factors_a/ (accessed March 11, 2026).

[B41] Olesen T. (2025). “Digital opacity,” in The Sociology of Whistleblowing, eds. B. N. Jaworsky, S. Worschech, and J. C. Alexander (Cham: Palgrave Macmillan), 109–130. doi: 10.1007/978-3-032-03476-2_6

[B42] Pennycook G. Rand D. G. (2019). Fighting misinformation on social media using crowdsourced judgments of news source quality. Proc. Natl. Acad. Sci. USA. 116, 2521–2526. doi: 10.1073/pnas.1806781116 30692252 PMC6377495

[B43] Petratos P. N. (2021). Misinformation, disinformation, and fake news: cyber risks to business. Bus. Horiz. 64, 763–774. doi: 10.1016/j.bushor.2021.07.012

[B44] Ramaul L. Ritala P. Kostis A. Aaltonen P. (2025). Rethinking how we theorize AI in organization and management: a problematizing review of rationality and anthropomorphism. J. Manage. Stud. 63, 761–807. doi: 10.1111/joms.13246

[B45] Ramirez A. (2007). The effect of anticipated future interaction and initial impression valence on relational communication in computer-mediated interaction. Commun. Stud. 58, 53–70. doi: 10.1080/10510970601168699

[B46] Sandra L. (2025). Digital emotional regulation paradox: a cross-sectional study on mindful technology use moderates the relationship between social media emotional content exposure and psychological resilience. BMC Psychol. 13:1411. doi: 10.1186/s40359-025-03727-4 41291860 PMC12751459

[B47] Sharma P. N. Liengaard B. D. Hair J. F. Sarstedt M. Ringle C. M. (2023). Predictive model assessment and selection in composite-based modeling using PLS-SEM: extensions and guidelines for using CVPAT. Eur. J. Mark. 57, 1662–1677. doi: 10.1108/EJM-08-2020-0636

[B48] Shrotryia V. K. Dhanda U. (2019). Content validity of assessment instrument for employee engagement. Sage Open 9, 1–7. doi: 10.1177/215824401882175134290901

[B49] Tarafdar M. Pullins E. B. Ragu-Nathan T. S. (2015). Technostress: negative effect on performance and possible mitigations. Inform. Syst. J. 25, 103–132. doi: 10.1111/isj.12042

[B50] Tidwell L. C. Walther J. B. (2002). Computer-mediated communication effects on disclosure, impressions, and interpersonal evaluations: getting to know one another a bit at a time. Hum. Commun. Res. 28, 317–348. doi: 10.1111/j.1468-2958.2002.tb00811.x

[B51] Vaccari C. Chadwick A. (2020). Deepfakes and disinformation: exploring the impact of synthetic political video on deception, uncertainty, and trust in news. Soc. Media Soc. 6, 1–13. doi: 10.1177/2056305120903408

[B52] van der Linden S. Roozenbeek J. Compton J. (2020). Inoculating against fake news about COVID-19. Front. Psychol. 11:566790. doi: 10.3389/fpsyg.2020.566790 33192844 PMC7644779

[B53] Vartiainen H. Liukkonen P. Tedre M. (2025). Emerging human-technology relationships in a co-design process with generative AI. Think. Skills Creat. 56:101742. doi: 10.1016/j.tsc.2024.101742

[B54] Walther J. B. (1992). Interpersonal effects in computer-mediated interaction: a relational perspective. Communic. Res. 19, 52–90. doi: 10.1177/009365092019001003

[B55] Wardle C. Derakhshan H. (2017). Information Disorder: Toward an Interdisciplinary Framework for Research and Policymaking. Strasbourg: Council of Europe. Available online at: https://scholar.google.com/citations?view_op=view_citationandhl=enanduser=Cil_o8UAAAAJandcitation_for_view=Cil_o8UAAAAJ:WF5omc3nYNoC (accessed March 25, 2026).

[B56] Yaşlioglu M. M. (2017). Sosyal Bilimlerde Faktör Analizi ve Geçerlilik: Keşfedici ve Dogrulayici Faktör Analizlerinin Kullanilmasi. Ist. Üniv. Işlet. Fak. Derg. 46, 74–85. Available online at: https://dergipark.org.tr/en/pub/iuisletme/article/357061

[B57] Yildirim A. Yolcu E. (2022). Sahte ne kadar derin? derin sahte (deepfake) kavraminin izini youtube üzerinden sürmek. Elektronik Cumhuriyet Iletişim Derg. 4, 63–74. doi: 10.54089/ecider.1110865

[B58] Zhao D. Wang Y. Zhang Q. Zheng R. Wu Y. J. Shu E. . (2026). The impact of digital orientation on digital product innovation: the mediating role of organizational unlearning. J. East Eur. Manage. Stud. 31:44619. doi: 10.31083/JEEMS44619

